# Hospital Sunday Fund

**Published:** 1889-06-22

**Authors:** 


### Hospital Sunday Fund.

### SIR ANDREW CLARK PLEADS FOR THE HOSPITALS.

Sir Sidney Waterlow presided at the meeting held in the Mansion House on Wednesday in connection with the Metropolitan Hospital Sunday Fund, the Lord Mayor being, unfortunately, seriously indisposed. Sir Sidney referred, in a few well-chosen words, to the increasing necessities of the Metropolitan hospitals, and the unfortunate fact that the demands upon their resources were also growing. The improved scientific methods of treatment now in use, moreover, while more effective, were also more expensive. Over 2,000 beds were unoccupied in London, not because there was any lack of applicants for admission, but because the governors of the institutions were insufficiently provided with funds. He considered, after an experience of sixteen years, during which time he had been vice-president of the Hospital Sunday Fund, that three or four hundred beds was the most that should be vacant in the metropolis.

Sir Andrew Clark then moved the first resolution—viz.: "That this meeting pledges itself to use every endeavour to impress upon the inhabitants of London the importance of maintaining the hospitals and medical charities in the utmost efficiency. In furtherance of this object it urges the clergy and the ministers of religion, in making their appeal for the required £100,000, to point out the advantage of giving through the Hospital Sunday Fund, on account of the nominal cost of so collecting funds for the hospitals." In these days we heard a great deal said, and sometimes very boastfully, of the triumphs of civilisation; and undoubtedly those triumphs were many and great. It would not be quite so interesting, though it might be a little more instructive, to hear a little about the failures of civilisation. And if one were to stand outside a metropolitan hospital and watch the people who entered, he would soon discover one of the failures. The sad, pale, sick countenances of those people were the result of one of the failures of civilisation. For the advances we were making also made it more difficult for men to live; it rendered necessary a constant effort to keep pace with others and to preserve health; and the very success with which medical research had been attended, added to these difficulties. It was true that an increasing knowledge of sanitary medicine helped to counteract them, but our capacity for adjusting things was pretty nearly at an end; whilst advancing civilisation is still developing new forms of accident and disease. People were wanted, therefore, to make such matters their study, and they were called doctors, of whom it could not be said—as with poets—that they were "born, not made." They had to be made, so how were we to give doctors the experience necessary to enable them to meet and deal with these cases? There was only one way, and that was to educate, train, and discipline them in hospitals, where they would be taught to exercise their crafts. Traditions and books were worthless, unaccompanied by practice; and, moreover, hospital work was necessary because no one could learn his profession from visiting single cases. Diseases must be watched, and their nature recorded in numbers, if a practical knowledge of them was to be acquired. So that if we suffer from disease we must have doctors, and if we have doctors we must also have hospitals. This would be evident when they considered what a hospital really is, consisting, as it does, of two departments: (1) the executive administration and (2) the medical working. The first involved buildings, wards, and necessaries; each ward being almost a miniature hospital, containing every provision for receiving sick persons, every appliance for heating, ventilation, nursing, and good attendance. Then there were young men to watch the development of each case; matrons to superintend the nursing and the management, and doctors and surgeons ready to visit and do their best for the relief of the sufferers. The second department, the medical, had its clinical clerk—who took the history of each case—an advanced student, assisted by the house surgeon, who in his turn brought the young students to it and explained how he intended to treat the case. Prescriptions were then made and administered, the physician again visiting the patient in three days' time, when the lesson would be continued. All these interesting methods were carried out day by day in the truly great hospitals, and they constituted their primary and fundamental objects. But an immense amount of collateral work was involved, which, though secondary, was scarcely less important. For instance, there was (1) the training of nurses, (2) the training of young doctors—for here alone could they acquire knowledge, (3) the perfection of surgeons, for daily practice enabled them to see what modes of treatment were undesirable, and so neglect them, (4) the discovery of new methods and of new medicines. And the results of all this work were not confined to the students and nurses and patients; for the nurses and doctors would take their knowledge, so acquired, and carry it with them to the utmost parts of the civilised world for the behoof of mankind in every region. There were certain other services which these hospitals rendered: They afforded an immediate refuge to all cases of sudden illness. When disease came suddenly, it was of supreme importance that it should be attended to at once. In London, if a man was really ill, and this especially applied to diseases of an infectious nature, no other certificate or qualification was necessary to gain him admittance to a hospital. Another service they rendered was to obviate the necessity for the very poor seeing their loved ones suffering without being able to render assistance, and this especially applied to sickness in the hovels of East London, where the atmosphere and surroundings were extremely inimical to recovery. The community was apt to forget this advantage. Another service was that which our hospitals render to the sick of the working classes, who, though they were not so unhappily placed as the others, were seldom provided with the requisite comforts and attendance, besides being taken from their task of winning bread. It might be said with regard to these that our hospitals took them from surroundings the most unfavourable, and placed them straightway into those most favourable; for they removed the patients from a vitiated atmosphere, from the doubtful and infrequent visits of the charity doctor, and from a host of other evils which contribute to keep him in sickness. Immediately it became known that anyone was so placed, the hospitals were thrown open to him, and he passed from a purgatory into a paradise. But, unfortunately, while cases of this sort were increasing, the wards of the hospitals were not furnished with sufficient means of assisting them. The hospitals also rendered services to the rich, for from them the rich get their trained nurses and skilful doctors, and all the accumulated knowledge which these hospitals collect and correct and purify for mankind. As an instance of the value and results of the work done in hospitals, he could refer to many operations which were now performed, and which were evidence of a wonderful advance in surgical science. Thus, the operation of ovariotomy—*i.e.*, the removal of a growth from the stomach—had been recently performed no less than sixty-six times by one physician, without a single fatality, whereas only 30 years ago it used to be considered too dangerous to be attempted, and he who did so was considered to be seeking a false reputation at an unjustifiable risk to his patient's life. In the National Hospital for the Paralysed and Epileptic, not long ago, a man was admitted suffering from convulsions and fits, due to a growth on the spinal cord. This was removed by a skilful surgical operation, and the patient was now well: a feat that never would have been so much as dreamed of 25 years ago. Another remarkable surgical operation recently brought to his knowledge was the cutting away of eighteen inches of a patient's intestines, sewing the several ends together, and allowing them to join again. The man was walking about at his duty six weeks after the operation, quite well and healthy. Sir Andrew then drew attention to the vast number of patients that had been treated in the London hospitals during the year, the in-patients amounting to 71,000, and the out-patients to 1,500,000. This was a great work, and it had practically been done for nothing, and at a cost of self-denial, and peril to life which many could not realise. Only recently had they lost a young physician at Guy's Hospital, who had studied and investigated with unusual and noble assiduity, though he had probably not earned more than £200 in many years. He had met with his reward at last — the crown of martyrdom at thirty-four years of age! What would happen if all these hospitals were suddenly extinguished? What happened in towns and cities where there were none? They were ravaged by plagues and pestilences. And if London had no places of refuge, infectious diseases would spread like wildfire, and the town would be ablaze with them. But owing to the proper treatment which could now be employed, antiseptic treatment was possible, and thus blood-poisoning, erysipelas, and other complications could be avoided. It was almost certain that if these institutions were wiped out, not only would the great city be speedily desolated, but we should have no nurses and no doctors to tend the sick. It was no abuse of language to say that hospitals were the most precious of all our modern institutions, and they had to recognise the fact that whilst the incomes of the hospitals were diminishing, the demands on their assistance were increasing. There were a great number of reasons for this: (1) The diminution in their receipts from invested capital, owing to agricultural depression; (2) the diminution in the strength and the virtue of giving, for there was a time when a larger percentage of people gave substance and services—this virtue was falling into a weakly state; (3) the founding and maintenance of improper and unneeded hospitals, which robbed the more deserving of what they would otherwise receive. Sir Andrew gave an amusing hypothetical instance of this,* suggesting the case of a physician, who, unsuccessful in practice, turned his attention to the study of the big toe and its ailments, exaggerating their importance and setting up an intimate relationship between it and every part of the body. A circle of friends would then take the matter up, and establish a "Hospital for the Diseases and Ailments of the Toe," and draw away much of the support of the public. He deprecated such institutions. (4) The opposition of certain crotchetty, though well-meaning men, who interfered in many ways, and were only capable of construing everything in its worst light; seeing only what is wrong and faulty, and not recognising what is good. An instance of this was the opposition of one such well-meaning person to the treatment of out-patients. When thwarted, he attacked certain hospitals, depriving them of half the support they otherwise would have received, and the whole of the opposition arose from a mere mistake. He touched upon this matter with great regret; for some of the people he was obliged to criticise had the very best intentions. As an instance of the emptiness of some of their objections, he pointed out the extreme value of the out-patient departments. He could never have acquired a wide range of knowledge and practice himself but for the experience gained in the out-patient departments of the hospitals, where he had been able to come face to face with the first indications of disease. He opposed the contention that people should not be supplied with outdoor relief without paying for it. Many out-patients, though apparently well-to-do, could no better afford to pay for medical advice than the poorest artisans. He instanced a case of a clerk, who, though well-dressed, was, when questioned, found to be earning only 28s. a week, upon which he supported a sick wife and several children. The enquiry made at Manchester into the system resulted in the discovery that the proportion of out-patients tended at the infirmary who might have contributed something for their relief, was 8 per cent. of the whole number. That proportion was not great, and it was not common sense or reasonable to expect that any work could be carried on with perfection. Perfection of all work, indeed, was to be got out of its imperfections, and hospital work was no exception. What, then, was to be done? How were they to meet the difficulty of diminishing incomes and increasing demands? Two courses alone were open—(1) to increase their subscriptions; (2) to throw the maintenance of their hospitals on the rates. He hoped they would not go upon the rates. He admired the existing system of maintaining the institutions, because voluntary efforts promoted individuality, and that was a characteristic of Englishmen. Extreme civilisation was always tending to cast people into one mould and destroy individuality, which was, after all, the expression of God's will in man, which all true men ought to cultivate. Would they exchange all the advantages entailed in the voluntary support of these institutions, the love and the personal interest which ought to dominate all work of this kind, for rate-supported hospitals? He could not imagine it! Rate-supported hospitals could never compare with others, and they were sometimes extravagant, corrupt, and inefficient. He could not believe they would sacrifice the present institutions for dull, lifeless, cold, and mechanical hospitals supported by the rates! The only alternative was for the public to increase their subscriptions, and he believed that when the question was considered from a moral and physical standpoint, and when the inexpressible value of the hospitals was realised, they might depend that money would come in abundance. He emphasised the wide influence exerted by the hospitals. Everyone benefited by them: old and young, rich and poor. They lived in an atmosphere of the good which these hospitals do. And what returns were they to render for such inexpressible blessings? They ought to increase their subscriptions and personal services. He knew it would be urged that the incomes of the public were themselves diminishing, whilst they, too, were confronted with increasing expenditure and growing taxes, and a harder struggle for existence. That was true enough from their point of view. But from his point of view, these were the very reasons the public should give more. And he believed that if they did so, they would discover that their simple giving would be transfigured into a divine charity, and would fill the hearts of all who give and receive, alike, with life and gladness.

* For the full account see "The Hospital" for Nov. 20, 1886, p. 122, Vol. I.

---

Bath has kept Hospital Saturday for the first time with such good result that we are sure it will not be the last time. The coins collected were 5,940 pennies, 1,668 half-pennies, 144 farthings, 200 two shillings, 125 half-crowns, 1,140 shillings, 1,758 sixpences, 1,003 threepenny pieces, 17 sovereigns, 32 half-sovereigns, cheque for £1 1s., and nine £5 notes in lieu of copper. This was a much larger sum than was anticipated, and we tender Bath our hearty congratulations on having aroused such interest in the hospital question in its midst.

